# Scapula kinematics of pull-up techniques: Avoiding impingement risk with training changes

**DOI:** 10.1016/j.jsams.2015.08.002

**Published:** 2016-08

**Authors:** Joe A.I. Prinold, Anthony M.J. Bull

**Affiliations:** Department of Bioengineering, Imperial College London, UK

**Keywords:** Repeatability, Shoulder, Biomechanics, Skin-fixed scapula tracking, Kipping, Supraspinatus

## Abstract

**Objectives:**

Overhead athletic activities and scapula dyskinesia are linked with shoulder pathology; pull-ups are a common training method for some overhead sports. Different pull-up techniques exist: anecdotally some are easier to perform, and others linked to greater incidences of pathology. This study aims to quantify scapular kinematics and external forces for three pull-up techniques, thus discussing potential injury implications.

**Design:**

An observational study was performed with eleven participants (age = 26.8 ± 2.4 years) who regularly perform pull-ups.

**Methods:**

The upward motions of three pull-up techniques were analysed: palms facing anterior, palms facing posterior and wide-grip. A skin-fixed scapula tracking technique with attached retro-reflective markers was used.

**Results:**

High intra-participant repeatability was observed: mean coefficients of multiple correlations of 0.87–1.00 in humerothoracic rotations and 0.77–0.90 for scapulothoracic rotations. Standard deviations of hand force was low: <5% body weight. Significantly different patterns of humerothoracic, scapulothoracic and glenohumeral kinematics were observed between the pull-up techniques. The reverse technique has extreme glenohumeral internal–external rotation and large deviation from the scapula plane. The wide technique has a reduced range of pro/retraction in the same HT plane of elevation and 90° of arm abduction with 45° external rotation was observed. All these factors suggest increased sub-acromial impingement risk.

**Conclusions:**

The scapula tracking technique showed high repeatability. High arm elevation during pull-ups reduces sub-acromial space and increases pressure, increasing the risk of impingement injury. Wide and reverse pull-ups demonstrate kinematics patterns linked with increased impingement risk. Weight-assisted front pull-ups require further investigation and could be recommended for weaker participants.

## 1 Introduction

Pull-ups are a common training activity for a range of sports. A link between scapula kinematics and injury, most commonly shoulder impingement, is widely theorized and occasionally tested,^1^, ^2^ particularly in overhead activities. Shoulder impingement is the compression of the rotator cuff and subacromial bursa on the anterioinferior aspect of the acromion coracoacromial ligament.^3^ This can occur with extreme internal glenohumeral (GH) rotation during unloaded abduction and forward flexion.^4^

Anecdotal evidence indicates that reverse pull-ups are easiest to perform, while wide-grip pull-ups are implicated with higher incidences of shoulder pathology. Climbing and gymnastics, which utilize pull-up-like techniques, are strongly linked to shoulder pathology—particularly shoulder impingement.^5^, ^6^ However, there is no quantitative discussion of the scapula and upper limb kinematics, or comparisons of the many different techniques, for pull-ups.

Difficulties in measuring 3-D scapula kinematics, due to skin artefacts, contributed to the lack of quantitative literature. Non-invasive skin-fixed devices with multiple attachment points and optimal calibration have reduced errors at high angles of humeral elevation and throughout the ROM in dynamic tasks.^7^, ^8^

Pull-ups are a closed-chain activity; good motion repeatability is therefore theorized across the experimental group (inter-participant), allowing comparison of group averages. Large muscle contractions in the shoulder have been hypothesized to reduce the consistency of observed joint kinematics.^9^ Pull-ups will provide a challenging environment in which to observe the intra-participant repeatability.

The aim is to present a kinematics dataset that compares the humerothoracic, scapulothoracic, and glenohumeral rotations across three pull-up techniques and discuss potential injury risks associated with these techniques.

## 2 Methods

A convenience sample of eleven healthy male participants with no history of shoulder pathology participated (age = 26.8 ± 2.4 years, BMI = 22.2 ± 2.2 kg/m^2^, height = 1.80 ± 0.06 m). Participants were performing pull-ups as part of a regular training regime (>3 years training experience). The local ethics committee approved this study.

**Fig. 1:**
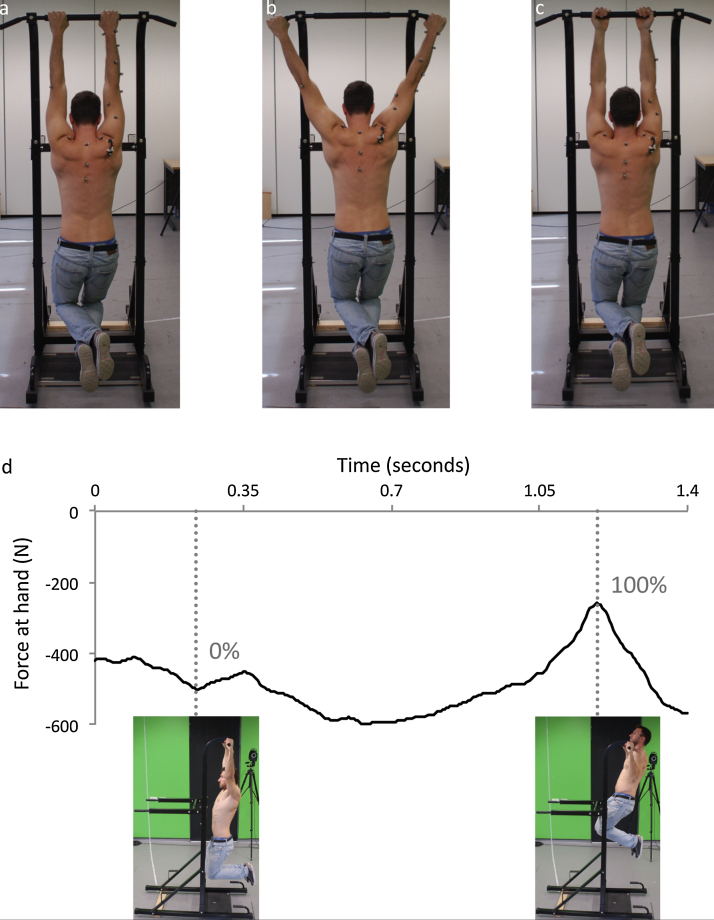
Experimental set-up showing position of the pull-up frame, force plate and participant. The three pull-up techniques are described: front (a) wide (b) and reverse (c), with the prescribed leg position. Normalization of the data is shown with force at one hand during a pull-up: 0% and 100% of the motion are marked (d). Images illustrate approximate body position at these two points for a representative participant.

Kinematic data collection utilized 9-camera optical motion tracking (Vicon, UK) at 200 Hz and a force plate (Kistler, Switzerland) at 1000 Hz (Fig. 1). A Scapula Tracker (ST^7^) measured scapula kinematics. The device consists of a base attached to the mid-portion of the scapula spine and an adjustable foot positioned on the meeting-point between the acromion process and the scapula spine. This position is optimal for the attachment of the ST.^8^ The ST technical coordinate frame was calibrated with the anatomical coordinate frame of the scapula using the International Society of Biomechanics (ISB) recommended anatomical landmarks^10^ and measured directly using a scapula Locator.^8^ Calibration was performed at 90° of humerothoracic (HT) elevation at 45° to the coronal plane: the mid-point of the overall motion.^7^ The calibration transformation was applied to each trial of that participant. Errors associated with static palpation of landmarks are small (∼2°^11^).

Twenty-one retro-reflective markers were used to track the thorax, clavicle, humerus and forearm.^8^, ^10^ Elbow epicondyles were defined as a rigid offset from the humerus technical frame with the arm at 90° elevation, 45° from the coronal plane, 90° elbow flexion and a vertical forearm. Least squares sphere-fitting was used without bias compensation^12^ to calculate the glenohumeral head centre during a functional task with low arm elevation, using the Locator to track the scapula.

Three pull-up techniques were performed: ‘front’ with anterior facing palms and hands approximately shoulder-width apart, ‘wide’ with anterior facing palms and hands on the lateral sloped portion of the bar and ‘reverse’ with posterior facing palms and hands approximately shoulder-width apart (Fig. 1). The hand positions were not prescribed between participants.

Five sets of three pull-ups were performed: each set was a random distribution of the techniques, giving five repetitions of each technique. Thirty-seconds rest was enforced between each set. Participants were instructed to perform a maximal upward movement covering their full range of motion (ROM), keeping legs to the posterior at 90° to the thorax (Fig. 1). The upward motion and the mean of three complete trials (randomly selected) for each participant were analysed.

Intra-participant repeatability is presented with coefficients of multiple correlations (CMC^13^) and standard deviations (SD). CMC is a measure of waveform similarity and has been used in gait analysis^14^ and shoulder kinematics.^13^ Inter-participant repeatability is calculated with Pearson's product moment coefficient of correlation (Pearson's *r*), because variables are centred and scaled according to their own means and standard deviations, thus waveform similarity is not sensitive to offsets in joint rotations expected with different participant anatomies. The average rotations for each participant are presented with an average inter-participant Pearson's *r* value for the three techniques.

A low-pass fourth-order Butterworth filter (cut-off: 4.7 Hz) was used to remove noise from the kinematics data. The ISB recommended coordinate frames were used for the thorax, humerus and scapula.^10^ ISB recommended Euler rotations were used, except at the GH joint where gimbal lock was observed and thus a *z*–*x*′–*y*″ sequence (adduction–flexion–internal rotation being positive) was used instead. A *z*–*y*′–*x*″ sequence (posterior tilt positive around *z*-axis) was used between the laboratory and thorax frames to determine thorax posterior tilt.

A low-pass fourth order Butterworth filter (cut-off: 10 Hz) was used on the force plate data, after a spectral analysis of the signal. To compensate for the lack of a second force plate, the vertical force when the participant is hanging from the bar minus the vertical force when the participant and frame are on the force plate is subtracted from that participant's trial's vertical force. Half the force values, normalized to the participant's body weight, give the force at each hand.

Data were normalized to the time of force time-points: zero percent was taken as the first peak in upward vertical reaction force occurring as the movement is initiated, 100% of the motion was taken as the major trough in this force (Fig. 1). A cubic spline interpolation was used to find the value of each measure at every 10% of the motion.

A two-way repeated measures ANOVA tested for significant differences between the three pull-up techniques (SPSS). Pull-up technique (front, wide, reverse) and percentage of motion (0–100%) were defined as the within-participant factors and joint rotations as the dependent variables. Where a significant interaction existed between technique and percentage of motion, a one-way repeated measures ANOVA tested for significant differences between the three pull-up motions at each 10% of the motion; percentage of motion was the within-subject factor. A Bonferroni post-hoc test then performed pair-wise comparisons between the techniques. Mauchly's test for sphericity was used. When a significant violation of sphericity was found the Greenhouse–Geisser correction was used. The Shapiro–Wilk test verified that the quantitative variables did not significantly depart from a normal distribution.

## 3 Results

Mean intra-participant repeatability is presented for each joint rotation and each pull-up technique (Table 1). CMC and Pearson's *r* values below 0.4 represent poor reliability, above 0.75 excellent reliability and between 0.4 and 0.75 fair to good reliability.^15^ The presented intra-participant variations are excellent; with no average CMC below 0.77 (and most ≥0.87). The wide and reverse pull-ups show the worst CMC values in ScT rotations (in protraction) and the reverse pull-up for HT rotations (in axial rotation). The force produced at one hand for all participants and motions had a maximum intra-participant SD of less than 5% body weight.

**Table 1:** Intra- and inter-participant repeatability of humerothoracic rotations (HT), scapulothoracic rotations (ST), thorax tilt (TH) and vertical hand force (FORCE) across three trials in three pull-up techniques. For intra-participant variations, mean values are presented across all participants ± standard deviation, and coefficients of multiple correlation (CMC) and standard deviation (SD) are used. For inter-participant variations mean Pearson's *r* values are presented ± standard deviation, with percentage of values that were significant (*p* < 0.05). Mean standard deviations are also presented across the eleven participants (SD). Humerothoracic rotations are plane of elevation (PoE), elevation (elev) and axial rotation (axial). Scapulothoracic rotations are lateral rotation (lat), protraction (pro) and posterior tilt (tilt). The range of the CMC values are zero (no relationship) to one (purely linear relationship).

Intra-participant repeatability						
	Front		Wide		Reverse	
**HT**	CMC	SD	CMC	SD	CMC	SD
PoE	0.96 ± 0.05	5.32 ± 1.50	0.95 ± 0.05	5.66 ± 1.90	0.90 ± 0.14	4.97 ± 1.97
Elev	0.98 ± 0.02	4.00 ± 2.04	0.99 ± 0.01	3.25 ± 1.56	1.00 ± 0.01	2.85 ± 1.18
Axial	0.96 ± 0.03	3.60 ± 1.86	0.94 ± 0.04	3.24 ± 1.04	0.87 ± 0.16	3.44 ± 1.22

**ST**						
Lat	0.98 ± 0.02	1.88 ± 0.72	0.98 ± 0.01	1.64 ± 0.74	0.98 ± 0.02	1.66 ± 0.94
Pro	0.90 ± 0.08	2.51 ± 1.08	0.77 ± 0.19	2.44 ± 1.36	0.83 ± 0.17	2.35 ± 0.96
Tilt	0.84 ± 0.16	1.33 ± 0.44	0.85 ± 0.12	1.81 ± 0.64	0.85 ± 0.10	1.36 ± 0.56

**TH**						
Tilt	0.89 ± 0.04	3.47 ± 1.45	0.91 ± 0.09	2.94 ± 0.80	0.92 ± 0.06	2.83 ± 1.57

**Force**						
Vertical	0.95 ± 0.03	2.40 ± 0.70	0.95 ± 0.03	2.22 ± 0.97	0.91 ± 0.08	2.71 ± 1.26

Inter-participant repeatability									
	Front			Wide			Reverse		
**HT**	Pearson's *r*	*p* < 0.05	SD	Pearson's *r*	*p* < 0.05	SD	Pearson's *r*	*p* < 0.05	SD
PoE	0.98 ± 0.02	100%	21.7	0.96 ± 0.04	100%	18.2	0.92 ± 0.07	100%	20.5
Elev	0.97 ± 0.03	100%	10.0	0.99 ± 0.01	100%	6.4	0.99 ± 0.01	100%	7.3
Axial	0.76 ± 0.32	85%	17.1	0.94 ± 0.06	100%	15.2	0.64 ± 0.36	91%	17.9

**ST**	Pearson's *r*	*p* < 0.05	SD	Pearson's *r*	*p* < 0.05	SD	Pearson's *r*	*p* < 0.05	SD
Lat	0.98 ± 0.02	100%	11.6	0.97 ± 0.03	100%	10.2	0.95 ± 0.06	100%	8.6
Pro	0.84 ± 0.13	100%	7.3	0.38 ± 0.44	82%	6.9	0.78 ± 0.16	100%	8.1
Tilt	0.622 ± 0.28	85%	6.9	0.66 ± 0.23	96%	7.8	0.55 ± 0.32	89%	6.8

**TH**	Pearson's *r*	*p* < 0.05		Pearson's r	*p* < 0.05		Pearson's *r*	*p* < 0.05	
tilt	0.771 ± 0.25	95%	–	0.60 ± 0.42	93%	–	0.79 ± 0.21	98%	–

**FORCE**	CMC	–		CMC	–		CMC	–	
	0.66	–		0.65	–		0.64	–	

The excellent Pearson's *r* values describe a clear trend in the measured values for the three pull-up techniques (Table 1). However, the posterior tilt during the reverse technique and, more significantly, the protraction during the wide technique show relatively poor correlations (Table 1) indicating differing scapula rotations and control between participants.

**Fig. 2:**
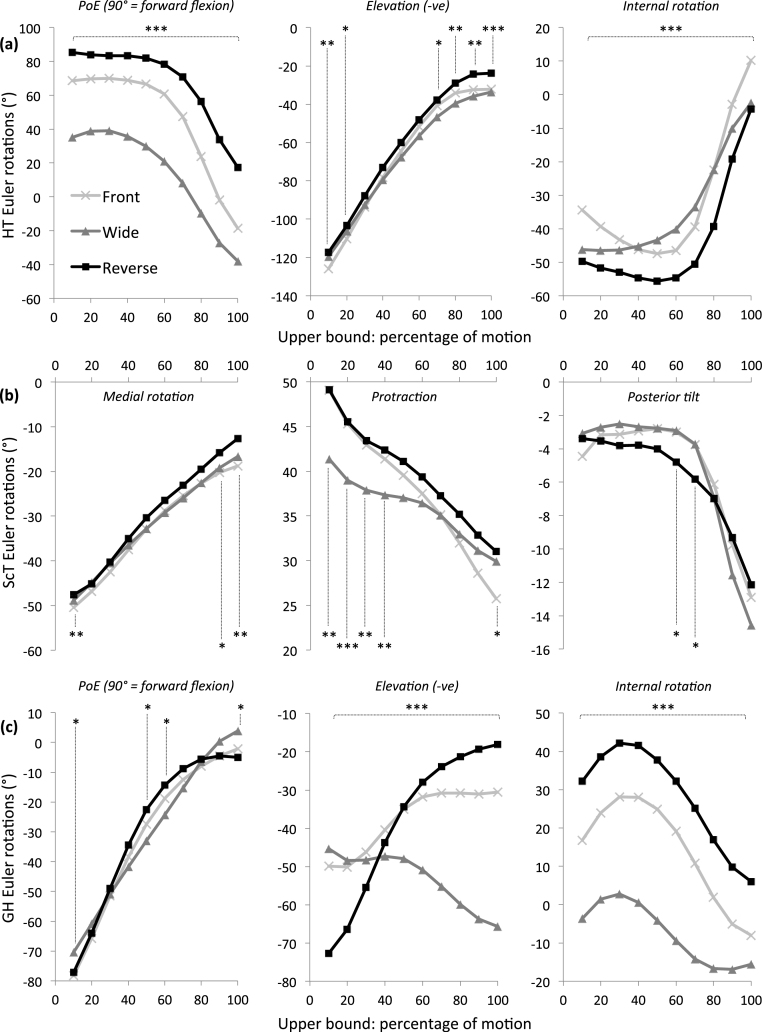
Mean Euler rotations for; (a) humerothoracic (HT) plane of elevation, elevation, and axial rotation; (b) scapulothoracic (ScT) medial rotation, protraction, and posterior tilt; (c) glenohumeral (GH) plane of elevation, elevation, and axial rotation during the front, wide and reverse pull-up techniques. Significant differences between all three motions are shown from a two-way repeated measures ANOVA test with pull-up technique and percentage of motion (0–100%) as the within-participant factors and HT, ScT, or GH rotations as the dependant variables. * Indicates *p* < 0.05, ** *p* < 0.01, *** *p* < 0.0001. N.B.: 0° HT/GH plane of elevation is abduction, 90° is forward flexion, and elevation is negative i.e. a more negative value indicates a more elevated arm. Otherwise, the named rotation is positive. Results of the one-way ANOVA testing and the Bonferroni post-hoc test are shown in the Supplementary material.

The average kinematics across the three pull-up techniques are presented (Fig. 2). The results of the one-way ANOVA testing, and the Bonferroni post-hoc tests, highlight where the differences between specific techniques are significant (Supplementary 1). The HT plane of elevation and axial rotation shows consistently significant differences between the three pull-up techniques (Fig. 2a). The most significant scapular differences are in scapula pro/retraction where front, wide and reverse pull-ups have ranges of 22°, 10° and 17°, respectively. Significant but small differences are seen in the other two rotations, with average ranges of 10° and 35° in medial/lateral rotation and ant/posterior tilt, respectively.

There was a significantly different pattern of GH internal/external rotation and plane of elevation between the three pull-up techniques (Fig. 2c).

## 4 Discussion

A novel dataset has been presented, describing shoulder kinematics during three pull-up techniques. There is no data in the literature with which to compare these results. In general, high elevation of the arm reduces sub-acromial space and increases pressure; thus increasing the risk of impingement.^4^, ^26^, ^29^

The HT rotations are within acceptable limits^22^ and, from qualitative examination, seem to describe the observed pull-up motions (Fig. 2). Significantly different planes of HT elevation and axial rotations result from different hand positions in the pull-up techniques (Fig. 1). More ScT retraction towards the top of the front pull-up, compared to the reverse pull-up, is expected because the humerus plane is more coronal during front pull-ups, which acts to retract the scapula. The more coronal plane of HT elevation at the bottom of the wide pull-ups (Fig. 2a) led to significantly more ScT retraction than the other two techniques. The greater ScT lateral rotation at the bottom of the front pull-up technique is expected with the increased humeral elevation. Similarly, there is a reduced ScT lateral rotation during the reverse pull-up, in-line with reduced HT elevation. ScT posterior tilt is similar between the three techniques. Given the small ROM it is unlikely that the ST is able to precisely differentiate between the techniques,^7^ although significant differences do exist (Fig. 2b). Overall, the pattern of ScT motion and the observed ROM is comparable to a bone-pin study of multi-planar humeral elevation.^22^

*Rotator cuff pathologies*, especially impingement are related to glenohumeral (GH) joint kinematics.^1^ Impingement is prevalent in climbers and gymnasts,^5^, ^6^ both requiring similar tasks to pull-ups. Therefore, speculation on vulnerable positions during pull-up techniques is a justified activity.

There is a significantly larger range of GH internal/external rotation in the reverse technique, starting in a position of quite extreme external rotation.^22^ Extreme external rotation with an elevated arm has been linked to impingement in athletic patients,^25^ high sub-acromial pressures^4^ and reduced sub-acromial space.^26^ Thus, the reverse pull up technique potentially increases sub-acromial impingement risk in the hanging and initiation phase, an important consideration, given that it is anecdotally easier and thus prescribed for weaker participants. Further work could analyse weight-assisted front pull-ups as a lower risk alternative.

In a cadaver study^4^ internal rotation of the humerus during abduction and flexion gave the highest supraspinatus compression forces. The limits of internal rotation^22^ are not observed in pull-up kinematics (Fig. 2c). However, during wide pull-ups 90° of arm abduction with 45° external rotation was observed (Fig. 2c). This position has been shown to give significantly smaller sub-acromial spaces than other abduction positions, although the acromion is not as close to the vulnerable part of the supraspinatus as in 45° of internal rotation.^26^ Greater protraction of the scapula relative to the humerus frame has also been shown to reduce sub-acromial space.^27^ A significantly reduced range of ScT pro/retraction (Fig. 2b), with a similar range of HT plane of elevation (Fig. 2a), in wide pull-ups may also increase sub-acromial impingement risk. Increased ScT ant/posterior tilt compensates somewhat, although the magnitude of this rotation is small. The wide pull-up may therefore be associated with an increased injury risk, a concern given the popularity of “Kipping” pull-ups (swinging and then performing a dynamic wide pull-up). The dynamic nature is likely to decrease scapula control, particularly in the starting position.^28^

Studies looking at sub-acromial space and pressure have been performed in unloaded, passive conditions or in cadavers. Thus, conclusions may be different to the highly loaded pull-ups presented here. However, given the position of the hand loading, the GH head is expected to be pulled more upwards onto the acromion.

The plane of elevation of the GH joint follows an expected pattern given the hand positions. The significant deviation of the humerus from the plane of the scapula during reverse pull-ups (up to 42°) and, to a lesser extent, front pull-ups (up to 28°), may require greater stabilization by the rotator cuff muscles, since the prime movers tend to move the GH reaction force outside of the glenoid rim in these poses. Modelling work could investigate these ideas.

Examination of *intra-participant repeatability* allows analysis of both movement and measurement method consistency. CMC values are sensitive to small differences in ROM, such as in ScT posterior tilt values. The associated SDs indicate whether there is large movement variability or statistical sensitivity.

The SD values for intra-participant repeatability of HT rotations were in-line with a palpation study of simple planar motions.^16^, ^17^ The CMC values were also excellent (>0.85). Thus variations can be considered small and participants to be consistently performing the same motion. The ScT rotations showed similar SDs to literature,^17^, ^19^, ^20^ and excellent CMC values (>0.75; Table 1) also in agreement with literature values.^19^, ^20^ The reduced repeatability in ScT protraction and posterior tilt may be due to lower accuracy of the ST in these rotations relative to lateral rotation.^7^ These results indicate that the ST has similar repeatability to accepted measurement techniques when applied to athletic loaded activities, although caution should be used in analysing posterior tilt.

The repeatability of the external force (Table 1) is important,^18^ as is participants’ posture. CMC values are excellent for both (>0.88), particularly external force (>0.9). SDs are also low relative to the parameter's magnitude (<5% for force). These indicate that each participant's motion and body acceleration profile was consistent between trials. Higher repeatability of forces relative to kinematics (Table 1), may imply pull-ups are a force-driven task.

*Inter-participant repeatability* must be good to allow kinematic data averaging across participants. High inter-participant variability (Table 1) relative to intra-participant values (Table 1) is expected.^17^

Large variations (SD up to 20°), of a similar scale to those observed in pull-ups (Table 1), exist in the HT plane of elevation and axial rotation during simpler and more controllable motions in the literature.^16^, ^17^ The generally good values of Pearson's *r* in HT rotations (0.64–0.99) also indicate that the pattern of the motion is repeatable between participants.

Inconsistent positioning of the hands may cause variation in HT rotations. Higher correlations observed in the wide technique, where the sloping pull-up frame handles determined the hand positions (Fig. 1), supports this. The position is consistent for each individual participant (Table 1), but enforcing a fixed hand position between participants would lead to unnatural, awkward or high-risk movements.

The ScT variability, measured by SD, is consistent with values found using established scapula tracking methods in the literature (7–12°^9^, ^17^). The values of Pearson's *r* are low in posterior tilt (0.55–0.66) and protraction during the wide pull-up (0.38), although not dissimilar to literature values.^9^ Pearson's *r* can be very sensitive to small differences where the range of movement is small, as in these rotations (Fig. 2). The implied difficulty in scapula control during the wide pull-up technique, shown by relatively poor intra-participant repeatability, may also help to explain this variation. However, given the good agreement with literature values the ScT inter-participant repeatability is considered acceptable for presentation of general kinematic patterns.

It is theorized that the scapula's starting position may be important in determining subsequent scapula rotations.^21^ This seems reasonable in highly-loaded activities, because it may be difficult and physiologically costly to move the scapula according to a ‘natural’ rhythm. In the current study, this may be manifested in higher intra-participant repeatability for force compared to kinematics (Table 1). Future investigation of scapula starting position's effect on kinematics and ‘natural’ scapula motion patterns would be valuable in the rehabilitation setting, where modification of scapula motion is implemented.^1^, ^20^ For pull-up training this potential relationship would imply that starting shoulder posture is key for injury prevention and targeting specific muscle groups. A setting of the scapula at rest and under loading has been theorized.^21^, ^24^ This may occur in the hanging phase of pull-ups: potentially causing significant scapula movement at the beginning of pull-ups, transitioning from a set position to a dynamic movement. Given that the start of the motion (0%) is taken as the initial peak in the hand force this is unlikely to be captured in the data presented. This dynamic process may contribute to the ST wobble observed in some participants.

The pattern of hand force and thorax tilt is similar across participants (Table 1). Un-loaded planar motions give a smaller range of ScT pro/retraction than literature values: ≈10°^22^ compared to ≈20° during pull-ups (Fig. 2). The force acting at the hand may pull the scapula around the thorax by means of muscular and ligamentous attachments, thus increasing ScT protraction.

Since general agreement exists in the inter-participant waveform patterns, performance of data averaging was valid.

A low cut-off frequency was used to reduce noise observed in the ST at the beginning of some trials. This noise was possibly caused by ST wobble during the pull-up's acceleration phase. This filtering had minimal effect on other joint rotations. The level of filtering is in-line with literature studies and recommendations.^23^ The low frequency noise may result, in part, from the moment produced by ST's marker cluster. Improved design would position markers at the ST's base.

Limitations of the study include the small sample size and a sample of convenience, which makes extrapolation to other populations somewhat limited.

## 5 Conclusion

A novel kinematics dataset has been presented. Potential links between wide and reverse pull-ups, and increased injury risk have been described. In reverse pull-up this is due to extreme external rotation with an elevated arm and a large deviation of the humerus from the scapula plane. In wide pull-ups it is due to a reduced range of ScT pro/retraction with a similar range of HT plane of elevation, and the achievement of 90° of arm abduction with 45° external rotation, and indicators of reduced scapula control.

## 6 Practical implications

- Novel kinematics data has been presented that describes the motion of the shoulder complex during a very commonly used training activity, which is relevant to a number of sports.
- Wide and reverse pull-ups demonstrate several kinematics patterns that are linked with increased shoulder injury risk (rotator cuff pathology such as impingement). The reverse technique has extreme glenohumeral internal–external rotation, and the wide technique has a reduced range of pro/retraction in the same HT plane of elevation—both potentially suggesting increased sub-acromial impingement risk.
- Since two of the techniques analysed appear to demonstrate kinematics patterns that are linked with injury risk, weight-assisted front pull-ups require further investigation and could be recommended by clinicians and personal trainers for those with risk factors and weaker participants.
- A scapula tracking technique showed high repeatability, in-line with literature values for accepted measurement techniques, during a dynamic activity. This adds to existing evidence that the method may be a repeatable way to clinically examine scapula kinematics.

## References

[1] Ludewig P.M., Reynolds J.F. (2009). The association of scapular kinematics and glenohumeral joint pathologies. J Orthop Sports Phys Ther.

[2] McClure P.W., Michener L.A., Karduna A.R. (2006). Shoulder function and 3-dimensional scapular kinematics in people with and without shoulder impingement syndrome. Phys Ther.

[3] Çalış M., Akgün K., Birtane M. (2000). Diagnostic values of clinical diagnostic tests in subacromial impingement syndrome. Ann Rheum Dis.

[4] Hughes P.C., Green R.A., Taylor N.F. (2012). Measurement of subacromial impingement of the rotator cuff. J Sci Med Sport.

[5] Caine D.J., Nassar L. (2005). Gymnastics injuries. Med Sport Sci.

[6] Rooks M.D., Johnston R.B., Ensor C.D. (1995). Injury patterns in recreational rock climbers. Am J Sports Med.

[7] Prinold J.A.I., Shaheen A.F., Bull A.M.J. (2011). Skin-fixed scapula trackers: a comparison of two dynamic methods across a range of calibration positions. J Biomech.

[8] Shaheen A.F., Alexander C.M., Bull A.M. (2011). Effects of attachment position and shoulder orientation during calibration on the accuracy of the acromial tracker. J Biomech.

[9] van Andel C.J., van Hutten K., Eversdijk M. (2009). Recording scapular motion using an acromion marker cluster. Gait Posture.

[10] Wu G., van der Helm F.C., Veeger H.E. (2005). ISB recommendation on definitions of joint coordinate systems of various joints for the reporting of human joint motion—Part II: Shoulder, elbow, wrist and hand. J Biomech.

[11] de Groot J.H. (1997). The variability of shoulder motions recorded by means of palpation. Clin Biomech.

[12] Lempereur M., Leboeuf F., Brochard S. (2010). In vivo estimation of the glenohumeral joint centre by functional methods: accuracy and repeatability assessment. J Biomech.

[13] Amasay T., Karduna A.R. (2009). Scapular kinematics in constrained and functional upper extremity movements. J Orthop Sports Phys Ther.

[14] Steinwender G., Saraph V., Scheiber S. (2000). Intrasubject repeatability of gait analysis data in normal and spastic children. Clin Biomech.

[15] Fleiss J. (1986).

[16] Prinold J.A., Villette C.C., Bull A.M. (2013). The influence of extreme speeds on scapula kinematics and the importance of controlling the plane of elevation. Clin Biomech.

[17] Meskers C.G., Vermeulen H.M., de Groot J.H. (1998). 3D shoulder position measurements using a six-degree-of-freedom electromagnetic tracking device. Clin Biomech.

[18] Kon Y., Nishinaka N., Gamada K. (2008). The influence of handheld weight on the scapulohumeral rhythm. J Shoulder Elbow Surg.

[19] Fayad F., Hoffmann G., Hanneton S. (2006). 3-D scapular kinematics during arm elevation: effect of motion velocity. Clin Biomech.

[20] Tsai N.T., McClure P.W., Karduna A.R. (2003). Effects of muscle fatigue on 3-dimensional scapular kinematics. Arch Phys Med Rehabil.

[21] Pascoal A.G., van der Helm F.F., Pezarat Correia P. (2000). Effects of different arm external loads on the scapulo-humeral rhythm. Clin Biomech.

[22] Ludewig P.M., Phadke V., Braman J.P. (2009). Motion of the shoulder complex during multiplanar humeral elevation. J Bone Joint Surg.

[23] Cooper R.A., DiGiovine C.P., Boninger M.L. (2002). Filter frequency selection for manual wheelchair biomechanics. J Rehabil Res Dev.

[24] Mottram S.L. (1997). Dynamic stability of the scapula. Manual Ther.

[25] Walch G., Boileau P., Noel E. (1992). Impingement of the deep surface of the supraspinatus tendon on the posterosuperior glenoid rim: an arthroscopic study. J Shoulder Elbow Surg.

[26] Graichen H., Bonel H., Stammberger T. (1999). Subacromial space width changes during abduction and rotation—a 3-D MR imaging study. Surg Radiol Anat.

[27] Solem-Bertoft E., Thuomas K.A., Westerberg C.E. (1993). The influence of scapular retraction and protraction on the width of the subacromial space. An MRI study. Clin Orthop Relat Res.

[28] Glassman G. (2014).

[29] Karduna A.R., Kerner P.J., Lazarus M.D. (2005). Contact forces in the subacromial space: effects of scapular orientation. J Shoulder Elbow Surg.

