# Supplementary material for: Scapula kinematics of pull-up techniques: Avoiding impingement risk with training changes
Source: J Sci Med Sport. 2016 Aug;19(8):629–35. doi: 10.1016/j.jsams.2015.08.002 (PMC4916995; doi:10.1016/j.jsams.2015.08.002)
Supplement: Supplementary file 1 [file mmc1.pdf]

		2-way	interaction		1-way	10°	20°	30°	40°	50°	60°	70°	80°	90°	100°
Humerothoracic	Plane of Elvation	***	**	F-W	***	***	***	***	***	***	***	***	**	*	-
				F-R	***	***	**	**	***	***	***	***	**	**	*
				W-R	***	***	***	***	***	***	***	***	***	***	**
	Elevation	*	**	F-W	-	-	-	-	-	-	-	-	-	-	-
				F-R	-	*	-	-	-	-	-	-	-	*	**
				W-R	**	-	-	-	*	*	*	*	**	***	***
	Internal rotation	***	-	F-W	-	n/a									
				F-R	***										
				W-R	**										
Scapulothoracic	Medial	*	*	F-W	-	*	*	-	-	-	-	-	-	-	-
				F-R	-	*	-	-	-	-	-	-	-	-	*
				W-R	-	-	-	-	-	-	-	-	-	-	-
	Protraction	-	***	F-W	-	***	***	**	*	-	-	-	-	-	*
				F-R	-	-	-	-	-	-	-	-	-	-	-
				W-R	-	**	*	*	-	-	-	-	-	-	-
	Posterior tilt	-	-	F-W	-	n/a									
				F-R	-										
				W-R	-										
Glenohumeral	Plane of Elvation	-	***	F-W	-	*	-	-	-	-	-	-	-	-	-
				F-R	-	-	-	-	-	-	-	-	-	-	-
				W-R	-	-	-	-	-	*	*	-	-	-	*
	Elevation	***	***	F-W	***	-	-	-	**	***	***	***	***	***	***
				F-R	-	***	***	**	-	-	-	*	**	**	**
				W-R	***	***	***	**	-	***	***	***	***	***	***
	Internal rotation	***	***	F-W	***	***	***	***	***	***	***	***	***	**	*
				F-R	***	**	**	**	***	***	***	**	**	**	**
				W-R	***	***	***	***	***	***	***	***	***	***	***

Supplementary 1: Significance testing results. For the three rotations tested, the overall results of the two-way repeated measures ANOVA ("2-way") are presented. The interaction between pull-up technique (Front, Wide, Reverse) and percentage of motion (0-100%) are presented ("interaction").The one-way repeated measures ANOVA results are also presented for all the combinations of comparison between the two pull-up types ("F-W" front-wide, "F-R" front-reverse, "W-R" wide-reverse). The results of the Bonferoni post-hoc tests are then presented for each 10% interval of the motion. * indicates $p<0.05$, ** $p<0.01$, *** $p<0.001$.
